# Twenty-four-hour ambulatory electrocardiography characterization of heart rhythm in *Vipera berus*-envenomed dogs

**DOI:** 10.1186/s13028-017-0296-x

**Published:** 2017-05-03

**Authors:** Anna Rave Vestberg, Anna Tidholm, Ingrid Ljungvall

**Affiliations:** 1 Anicura Regional Animal Hospital, Ljusnevägen 17, 12848 Bagarmossen, Sweden; 2 Anicura Albano Animal Hospital, Rinkebyvägen 21a, 18236 Danderyd, Sweden; 3 Department of Clinical Sciences, Faculty of Veterinary Medicine and Small Animal Sciences, Swedish University of Agricultural Sciences, P.O. Box 7084, 75007 Uppsala, Sweden

**Keywords:** *Vipera berus*, ECG, Ambulatory ECG, Dog, Arrhythmia, Snakebite

## Abstract

**Background:**

*Vipera berus* has a worldwide distribution and causes high morbidity in dogs annually. A complication to envenomation may be cardiac arrhythmias. The purpose of this study was to investigate the prevalence, types, and timing of arrhythmias, using 24-h ambulatory electrocardiography (24-AECG), in dogs bitten by *V. berus* in the first 24–32 h after envenomation. In addition, this study aimed to investigate if there were differences in selected clinical and hematological- and biochemical variables (including cardiac troponin I) at admission between *V. berus*-envenomed dogs with and without detected pathologic arrhythmias. Seventeen prospectively recruited client-owned dogs acutely envenomed by *V. berus,* were therefore examined clinically and echocardiographically, sampled for blood, hospitalized, and monitored by 24-AECG.

**Results:**

Clinically significant pathologic arrhythmias in this study were of ventricular origin, such as frequent single ventricular premature contractions (VPCs) and couplets of VPCs, episodes of ventricular tachycardia and idioventricular rhythm, and “R-on-T phenomenon”. Variations of these arrhythmias were detected by 24-AECG in eight (47%) of included dogs. No arrhythmias were detected by cardiac auscultation. Twenty-four hours following envenomation, four out of eight dogs experienced decreases (all *P* < 0.039), and three out of eight dogs experienced increases (all *P* < 0.034), in arrhythmic episodes. All four dogs bitten on a limb developed pathologic arrhythmias. Otherwise, no significant differences in clinical, hematological or biochemical variables were seen between dogs with pathologic arrhythmias and those without.

**Conclusion:**

Forty-seven percent of dogs bitten by *V. berus* included in this study experienced pathologic arrhythmias of abnormal ventricular depolarization. During the first 24–32 h from the snakebite, some dogs experienced a decrease in arrhythmic episodes and others an increase in arrhythmic episodes. These findings indicate a potential value of repeated or prolonged electrocardiography monitoring of envenomed dogs for identification of which dogs that might benefit the most from prolonged hospitalization for optimal monitoring and treatment of cardiac abnormalities. In the present study, dogs that developed arrhythmias could not be differentiated from dogs that did not based on clinical findings or hematological or biochemical variables obtained at admission.

## Background

Snakes of the Viperidae family, which has a worldwide distribution, yearly cause a significant number of envenomations in dogs [1–3]. *Vipera berus* is the only poisonous snake in Scandinavia and the United Kingdom [4]. The mortality rate of bitten dogs has been reported to vary from 0 to 7% [3, 5–8].

Most dogs are bitten on the nose or limbs and typical clinical signs include local swelling, edema and pain, as well as lethargy, depression, hyperthermia, salivation, and tachycardia [3, 5–7]. Various cardiac arrhythmias have been described as a complication to envenomation [9, 10]. Monitoring envenomed dogs for arrhythmias by auscultation is challenging because sustained supraventricular-, idioventricular-, or ventricular tachycardia frequently present as a regular rhythm [11]. Electrocardiography (ECG) is therefore required to diagnose rhythm disturbances. Few studies have investigated arrhythmias secondary to *V. berus* and *Vipera palaestinae* envenomation [9, 10, 12]. In a prospective study of 24 dogs bitten by *V. berus* examined by standard (short-time) ECG, six dogs presented with arrhythmias at the initial clinical examination and ten dogs had arrhythmias 12–24 h after admission [10].

However, studies have shown that in-hospital ECGs of 2–5 min are insensitive for detecting arrhythmias [13, 14] which suggests that the arrhythmic incidence reported in earlier studies of snake bitten dogs might be underestimated.

A study describing ECG abnormalities using continuous 24-h ambulatory electrocardiography (24-AECG) in horses bitten by rattlesnakes has been published [15], but, to the authors’ knowledge, 24-AECG studies investigating dogs bitten by viper snakes are lacking.

Abnormal hematological findings such as coagulopathy, neutrophilia, hemoconcentration, leukocytosis, and thrombocytopenia have been reported after envenomation by *V. berus* and *V. palaestinae* [1, 5–7, 12, 16, 17]. The reported biochemical abnormalities suggest mild multiple organ pathology, and elevated concentrations of muscle enzymes as a consequence of the envenomation [1, 5–7, 12, 16, 17].

Few studies have investigated circulating concentrations of cardiac specific troponin I (cTnI) in regards to envenomation by viper snakes. Concentrations of cTnI above reference ranges have been reported both at admission and 12–24 h after admission [9, 10, 12]. Increased cTnI concentrations do not seem to correlate with the presence of arrhythmias as some snake bitten dogs with arrhythmias have been shown to have normal concentrations of cTnI and vice versa [9, 10].

Increased knowledge of types of arrhythmias and when they potentially develop in relation to the envenomation episode would be beneficial to optimize monitoring and treatment strategies of dogs bitten by *V. berus*.

Therefore, the purpose of this study was to investigate the prevalence, types, and timing of arrhythmias, using 24-AECG, in dogs bitten by *V. berus* in the first 24–32 h after envenomation. In addition, we aimed to investigate the possible differences in selected clinical and hematological- and biochemical variables (including cTnI) at admission between *V. berus*-envenomed dogs with and without detected pathologic arrhythmias.

## Methods

The study was approved by the Local Ethical Committee of Animal Experiments (S56-13) and the Swedish Board of Agriculture (5.2.18-10320/13). All owners signed a written consent form prior to inclusion in the study.

Dogs bitten between March 2014 and June 2015, presented to Anicura Regional Animal Hospital, Stockholm, Sweden, were prospectively recruited to the study. To be included in the study, dogs had to exhibit typical clinical signs of envenomation (sudden pain, swelling, and edema) and the snake had to have been observed by the owner, either as it bit the dog or in the near proximity of the dog.

Dogs with primary cardiac disease (other than trivial mitral- or tricuspid insufficiency with normal valvular morphology), systemic disease or that were treated with any type of medication prior to arrival at the hospital were excluded from the study. In addition, dogs that were bitten more than 8 h before presentation were also excluded.

### Clinical evaluation and treatment strategy

Veterinary clinicians involved in the study had received oral instructions on how to examine the dogs and use the predesigned study protocol. Upon arrival at the animal hospital, dogs that met the inclusion criteria were examined by a veterinary clinician. The owners were interviewed about the time of the snake bite and the medical history of the dogs. Clinical findings with emphasis on the location of the snake bite, the appearance of the area of the bite (degree of swelling graded subjectively as no swelling or mild, moderate or severe swelling), and the dog’s cardiovascular and mental status (graded subjectively as normal or mildly, moderately or severely depressed), were recorded in the study protocol and in the dog’s medical record.

All dogs had a venous catheter placed in the distal cephalic vein and blood was sampled in EDTA, non-additive (serum), and heparin tubes, immediately after the clinical examination. The dogs were allowed short walks five times per 24 h. All dogs were treated according to the animal hospital’s treatment strategies of viper bites, and administered constant rate infusion of intravenous crystalloids^1^ and buprenorphine intravenously every eighth hour, at rates and doses at the discretion of the attending veterinary clinician. The morning after arrival, and every 24 h as long as the dogs were hospitalized, the clinical examination was repeated. The heart was auscultated every fourth hour by the attending veterinary clinician. It was the decision of the veterinary clinician in charge as to when the dog could be discharged. All dogs were recommended a revisit about 14 days after discharge from the animal hospital and the dogs were only permitted short leash walks until the revisit. At the time of the revisit, the owner was interviewed about any possible complications to the snakebite, the dog’s mental status and the appearance of the bite-area, and a clinical examination was performed. To investigate possible complications due to the snake bite, telephone interviews were conducted with the dog owners a minimum of 1 year after the snake bite.

### Laboratory analysis

Blood was collected for hematologic analyses in EDTA tubes and analyzed by an automated laser-based hematology analyzer.^2^ Blood smears were evaluated, and a manual differential count performed. Blood collected in serum tubes for biochemical analyses (C-reactive protein (CRP), glucose, urea, alanine amino transferase (ALAT), alkaline phosphatase (ALP), creatinine, albumin, total protein, total calcium, phosphate, bile acids, sodium, potassium, and chloride) was centrifuged (1500G, 5 min) 30 min after collection and analyzed by spectrophotometric methods.^3^ All the mentioned analyses were performed immediately after centrifugation.

Blood collected in heparin tubes for plasma cTnI analysis was centrifuged (1500*g*, 5 min) immediately after sampling and thereafter transferred to cryotubes, and stored in −20 °C for a maximum time of 3 months after which time the samples were transported in frozen form to −80 °C and stored for a maximum time of 29 months. Batched-analysis was performed with a commercially available high-sensitivity, enzyme-linked immunosorbent assay^4^, previously validated for use in dogs [18]. The lower detection limit of the assay was 0.01 ng/ml. All samples were analyzed in duplicate and the mean cTnI concentration for every dog was used for statistical analyses. The samples that contained a cTnI concentration lower than the detection limit of the assay was assigned a concentration of 0.005 ng/ml.

### Echocardiography

To rule out primary cardiac disease, the dogs underwent a standard two-dimensional echocardiographic examination^5^ immediately after the clinical examination. When this was impossible due to practical reasons, an echocardiographic examination was performed the following day or at the time of the revisit. The echocardiographic examinations were performed on unsedated and mildly restrained dogs in lateral recumbency. The left ventricular and right ventricular measurements were obtained from the right parasternal long and short axis views and the left atrial to aortic root ratio from the right parasternal short axis view, as previously described [19, 20]. Doppler examinations of all valves were performed and peak flow velocities were recorded.

### Electrocardiographic evaluation

The dogs were shaved behind the elbows and on the chest, and the skin was cleaned with alcohol. The electrodes^6^ were placed in a seven-lead precordial system and connected to a 24-AECG.^7^ The system was covered by a monitoring vest and set to record for 24 h while the dogs were hospitalized.

### Arrhythmia analyses

The recordings were analyzed by one of the authors (ARV) by a software^8^ designed for humans. All recordings were manually checked and adjusted. Only examinations of a minimum of 18 h of recordings were included in the study. The time of the snake bite was set as hour zero for every dog and the number of hours from the time of the bite to the start of the 24-AECG recordings were calculated to ensure correct comparison of the hourly summaries of the recordings between the different dogs. The electrodes and recorder were removed after 25 h unless the electrodes had not detached prematurely.

**Fig. 1:**
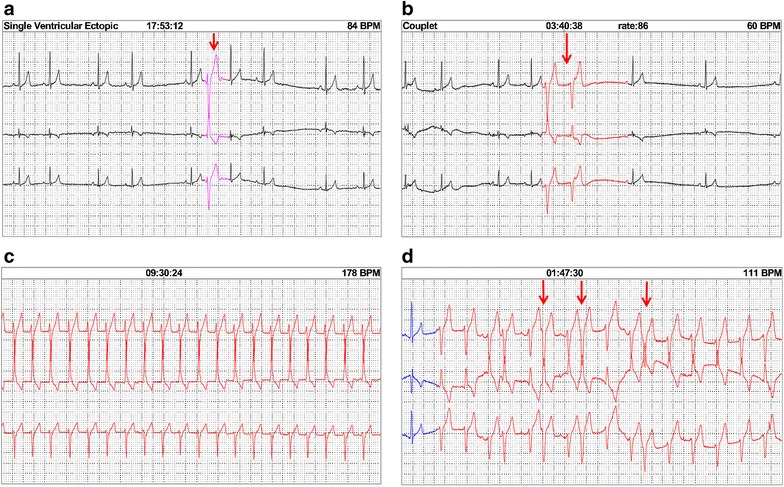
24-h ambulatory electrocardiography strips recorded from selected dogs envenomed by *Vipera berus* to illustrate some abnormalities of ventricular depolarization used to separate dogs with and without pathologic arrhythmias. Dogs with the following abnormalities were considered to have pathologic arrhythmias. **a** More than 50 single ventricular premature complex (VPC) (*arrow*). **b** Couplets of VPCs (*arrow*). **c** Episodes of ventricular tachycardia. **d** “R-on-T phenomenon” (*arrow*)

The 24-AECG recordings were reviewed according to the definitions presented in Table 1. Dogs were divided into two groups as follows: dogs with pathologic arrhythmias and dogs without pathologic arrhythmias, and the criteria for the division was based upon findings in previous published studies of 24-AECG in dogs [13, 21–24]. Dogs that had less than 50 single and sporadic ventricular premature complexes (VPCs) per 24 h were considered normal, and dogs that had more than 50 single VPCs per 24 h (Fig. 1a), couplets of VPCs (Fig. 1b), ventricular tachycardia (VT) (Fig. 1c) or “R-on-T phenomenon” (Fig. 1d) were considered to have pathologic arrhythmias.

**Table 1:** List of variables derived from the 24-h ambulatory electrocardiography and the respective definitions

Maximum, minimum, and mean heart rate during 24 h and for every hour
Total number of episodes of bradycardia [minimum of four consecutive sinus beats at the rate of <45 beats per minute (BPM)] during 24 h and for every hour
Total number of episodes of tachycardia (minimum of four consecutive sinus beats at the rate of >150 BPM) during 24 h and for every hour
Total number of sinus pauses (defined as the absence of sinus P waves for more than 2 s) in 24 h and for every hour
Total number of single atrial premature complexes (APCs) (defined as a premature normally appearing QRS complex with an abnormal P wave morphology) in 24 h and for every hour
Total number of couplets (two consecutive) of APCs in 24 h and for every hour
Total number of episodes of supraventricular tachycardia (SVT) (defined as an episode of ≥3 normally appearing QRS complexes with a rate of >150 BPM that commence with a complex of abnormal P wave morphology and whose N–N interval is equal to or less than the previous N–N interval) in 24 h and for every hour
Total time of SVT during 24 h
Total number of single ventricular premature complexes (VPCs) (defined as a premature complex with a bizarre and abnormally wide morphology and large T wave of opposite polarity) in 24 h and for every hour
Total number of couplets of VPCs in 24 h and for every hour
Total number of episodes of ventricular tachycardia (VT) (defined as an episode of ≥3 VPCs with at the rate of >100 BPM) in 24 h and for every hour
Total number of VPCs in episodes of ventricular tachycardia in 24 h and for every hour
Total time of VT in 24 h
Total number of idioventricular episodes (defined as an episode of >3 VPCs at a rate of <100 BPM) in 24 h and for every hour
Total number of idioventricular complexes (defined as a VPC at a rate of <100 BPM) in 24 h and for every hour
Total number of ventricular depolarizations (defined as the sum of single and couplet VPCs, number of VPCs in episodes of VT, and number of complexes in episodes of IVR) in 24 h and for every hour
Total number of episodes of “R-on-T phenomenon” (defined as an episode where a VPC interrupts the T wave of the preceding beat) in 24 h and for every hour
Presence of second-degree AV block (defined as a P wave not conducting a QRS complex)

*BPM* beats per minute, *APC* atrial premature complex, *SVT* supraventricular tachycardia, *VPC* ventricular premature complex, *VT* ventricular tachycardia, *AV block* atrioventricular block

### Statistical analysis

Statistical analyses were performed using a commercially available software program.^9^ A value of *P* < 0.05 was considered significant for the analyses. Group data such as dog characteristics, clinical findings, and arrhythmia variables are presented as medians and interquartile range (IQR). A logarithmic transformation was performed to achieve a normal distribution for skewed variables.

The non-parametric Mann–Whitney U test was used to compare continuous variables such as age, body weight, time from snake bite to admission and 24-AECG, duration of hospitalization, number of days from hospitalization to revisit, hematologic- and biochemical variables (including cTnI concentrations), and dose of buprenorphine, between dogs with pathologic arrhythmia and those without. The Chi square test was used for comparisons between categorical variables such as sex (female/male), area of bite (nose/limb), no/mild/moderate/severe swelling at admission and hospitalization, normal- and mildly/moderately/severely depressed mental status at admission, hospitalization, at home, and at revisit, and month of envenomation (subdivided into March–May and June–September) between dogs with pathologic arrhythmias and those without. To enable statistical analyses, no swelling and normal mental status were assigned 1, and mild(ly), moderate(ly), and severe(ly) swelling and depressed mental status were assigned 2, 3, and 4 respectively.

Univariate regression analyses were used to evaluate potential associations between hour after snakebite and the various ECG variables (listed in Table 1) and continuous and categorical variables described above for dogs with pathologic arrhythmias.

## Results

### Clinical findings

Breeds included Labrador retriever (n = 3), mixed breed (n = 3), German shepherd (n = 2), flat coated retriever (n = 1), Gonczy Polski (n = 1), Irish setter (n = 1), Jack Russel terrier (n = 1), Nova Scotia duck tolling retriever (n = 1), English pointer (n = 1), Rhodesian ridgeback (n = 1), Samoyed (n = 1), and Welsh springer spaniel (n = 1). Dog characteristics and clinical findings other than breed are summarized in Table 2.

**Table 2:** Dog characteristics and clinical findings for *Vipera berus*-envenomed dogs, and corresponding *P* values for group comparisons

Group	Dogs with pathologic arrhythmias	Dogs without pathologic arrhythmias	*P* value
Number	8/17	9/17	
Sex (female/male)	6/2	3/6	0.09
Age (years)	3.16 (IQR 1.62–4.34)	3.26 (IQR 1.02–5.45)	1.00
Weight (kg)	25.2 (IQR 20–29.9)	22 (IQR 17.6–36.4)	0.92
Month of bite (March–May/June–September)	4/4	5/4	0.82^a^
Area of bite (nose/limb)	4/4	9/0	0.02^b^
Time from bite to admission (hours)	1.85 (IQR 2.96–1.03)	2 (IQR 2–3)	0.89
Time from bite to 24-AECG (hours)	2 (IQR 1.25–3.75)	2 (IQR 2–3)	0.76
Mental status at admission (normal/mildly depressed/moderately depressed/severely depressed)	2/2/3/1 2.5 (IQR 1.25–3)	2/4/2/1 2 (IQR 1.5–3)	0.73
Degree of swelling of bite area at admission (normal/mildly/moderately/severely)	0/3/3/2 3 (IQR 2–3.75)	0/0/5/4 3 (IQR 3–4)	0.13
Mental status day after hospitalization (normal/mildly depressed/moderately depressed/severely depressed)	5/3/0/0 1 (IQR 1–2)	8/1/0/0 1 (IQR 1–1)	0.21
Degree of swelling of bite area day after hospitalization (normal/mildly/moderately/severely)	1/2/4/1 3 (IQR 2–3)	0/2/4/3 3 (IQR 2.5–4)	0.28
Mental status at home (normal/mildly depressed/moderately depressed/severely depressed)	8/0/0/0 I (IQR 1–1)	7/2/0/0 1 (IQR 1–1.75)	0.14
Mental status at revisit (normal/mildly depressed/moderately depressed/severely depressed)	7/0/0/0^b^ 1 (IQR 1–1)	7/0/0/0^c^ 1 (IQR 1–1)	1.00
Dose buprenorphine (mg/kg)	0.01 (IQR 0.006–0.012)	0.012 (IQR 0.011–0.013)	0.37
Arrhythmia or murmur detected with cardiac auscultation during hospitalization (yes/no)	0/9	0/9	
Additional clinical findings during hospitalization	Hematoma gingiva (n = 1) Edema limb (n = 1) Edema scrotum (n = 1) Diarrhea (n = 1)	Hematoma gingiva (n = 5)	
Additional medications during hospitalization	Maropitant (n = 1) Omeprazole (n = 1) Sucralfate (n = 2)	Maropitant (n = 1)	
Duration of hospitalization (days)	1.5 (IQR 1–2.75)	1 (IQR 1–2)	0.52
Time to revisit (days)	15 (IQR 11–17)	14 (IQR (13–16)	0.90
Concentration cTnI at admission	0.015 (IQR 0.006–0.039)^c^	0.01 (IQR (0.01–0.02)	1.00
Long-term complications to snake-bite (yes/no)^d^	0/8	0/5	

No swelling and normal mental status were assigned 1, and mild(ly), moderate(ly), and severe(ly) swelling and depressed mental status were assigned 2, 3, and 4 respectively. Dogs without pathologic arrhythmias had less than 50 single and sporadic VPCs per 24 h. Dogs with pathologic arrhythmias had more than 50 VPCs per 24 h, couplets of VPCs, VT or R-on-T. Group data is presented as medians and interquartile range (IQR)

^a^ *P* value for testing March–May/June–September by pathologic arrhythmia yes/no

^b^ *P* value for testing area of snakebite by pathologic arrhythmia yes/no

^c^seven dogs

^d^13 dogs (four dog owners could not be contacted). 24-h ambulatory ECG (24-AECG). Cardiac troponin I (cTnI)

### Echocardiography

Four dogs (24%) underwent echocardiographic examinations at admission, five (29%) dogs the day after admission, and eight (47%) dogs at the revisit.

All dogs had unremarkable echocardiograms except one dog with trivial mitral insufficiency.

### Electrocardiographic evaluation and arrhythmia analysis

All dogs had complete recordings of 24 h except three dogs that lacked 1, 2, and 6 h respectively. Clinically significant arrhythmias were of ventricular origin, and in the 24-AECG recordings, pathologic arrhythmias were found in eight of the 17 (47%) included dogs. Of the nine dogs with normal 24-AECG, none had more than seven ventricular depolarizations in total. None of the eight dogs with arrhythmias had less than 568 abnormal ventricular depolarizations. No dog had supraventricular arrhythmias other than single and sporadic atrial premature complexes (APCs). Sporadic events of second degree atrioventricular (AV) block was identified in four dogs in the group of dogs with pathologic arrhythmias and four dogs in the group of dogs without pathologic arrhythmias. A summary of dog characteristics and clinical findings in dogs with and without pathologic arrhythmias and *P* values obtained from the non-parametric Mann–Whitney U test and Chi square test are shown in Table 2.

Median and IQR for 12 selected 24-AECG variables of the eight dogs with pathologic arrhythmias are included in Table 3.

**Table 3:** Selected 24-h ambulatory electrocardiography variables derived from eight *Vipera berus*-envenomed dogs with pathologic arrhythmias

Variables 24-AECG	Median	IQR
Total number of QRS complexes	117,861	102,484–134,638
Maximum HR (BPM)	196	189–200
Minimum HR (BPM)	39.5	36–44.5
Average HR (BPM)	83.5	76.25–88.5
Single VPC	670	277–1626
Couplet VPC	155	55.5–416
Episodes of VT	200	33–331
Total number of VPCs in episodes of VT	3503	703–5720
Episodes of IVR	52	25.5–151
Total number of complexes in episodes of IVR	152	68–284
Total number of ventricular depolarizations	5241	1554–8059
Episodes of “R-on-T phenomenon”	0	0–4.8

Total number of ventricular depolarizations is defined as the sum of single and couplet VPCs, number of VPCs in episodes of VT, and number of complexes in episodes of IVR

*24-AECG* 24-h ambulatory ECG, *IQR* interquartile range, *HR* heart rate, *BPM* beats per minute, *VPC* ventricular premature complex, *VT* ventricular tachycardia, *IVR* idioventricular rhythm

### Characterization and timing of arrhythmias

**Fig. 2:**
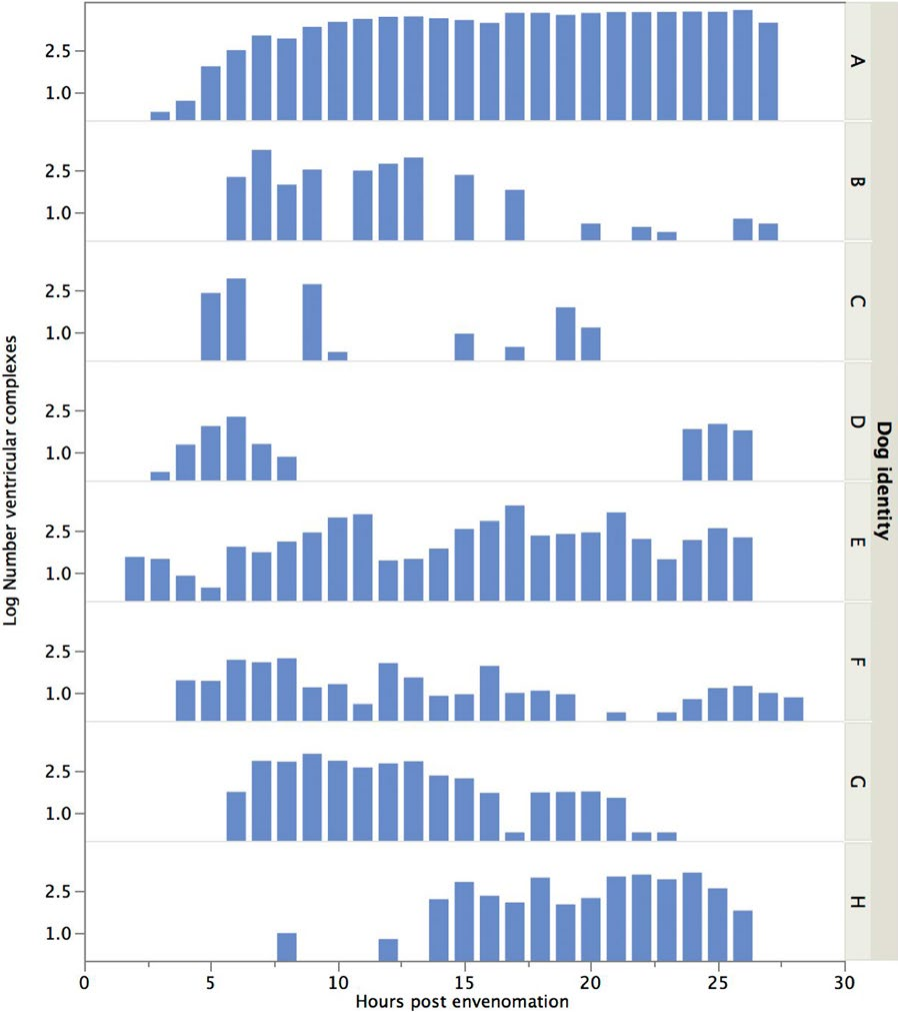
Total number of ventricular depolarizations in the first 24 h (+ number of hours from envenomation episode until start of 24-h ambulatory ECG recordings) after *Vipera berus* envenomation in eight dogs with pathologic arrhythmias. Dogs with identities *B* and *F* experienced a decrease (all *P* < 0.039) in total number of ventricular depolarizations. Dogs with identities *A*, *E*, and *H* experienced an increase (all *P* < 0.019) in total number of ventricular depolarizations. A logarithmic transformation of the total number of ventricular depolarizations was performed to achieve a normal distribution. *P* values are from the univariate regression models

Four of the eight dogs with pathologic arrhythmias experienced a decrease in episodes of arrhythmias in the 24 h following the snakebite. Two of these four dogs experienced a decrease (all *P* < 0.039) in each of the following categories: number of single VPCs, number of episodes of VT, total number of VPCs in episodes of VT, and total number of ventricular depolarizations (Fig. 2). One dog experienced a decrease in number of couplets of VPCs (*P* = 0.005, *R* ^*2*^ = 0.47). One dog experienced a decrease in the number of idioventricular episodes (*P* = 0.001, *R* ^*2*^ = 0.53), and one dogs had three episodes of “R-on-T phenomenon”, one episode occurring 7 h and two episodes occurring 13 h after the snake bite.

Three dogs with pathologic arrhythmia experienced an increase in episodes of arrhythmias in the 24 h following the snake bite. All three dogs experienced increases in the total number of ventricular depolarizations during the recorded 24 h (all *P* < 0.019) **(**Fig. 2). One dog experienced an increase in each of the following categories: number of single VPCs (*P* = 0.014, *R* ^*2*^ = 0.24), number of episodes of VT (*P* = 0.034, *R* ^*2*^ = 0.20), and total number of VPCs in episodes of VT (*P* = 0.0001, *R* ^*2*^ = 0.53). Two of the dogs had 13 and 27 episodes of “R-on-T phenomenon” respectively. No correlation between time and episodes of “R-on-T phenomenon” was found for the former dog but for the latter dog, there was a decrease in the number of episodes of “R-on-T phenomenon” (*P* = 0.017, *R* ^*2*^ = 0.53) during the 24 h following the snakebite.

### Laboratory analysis

Hematologic and serum biochemistry variables were within reference intervals for the majority of the dogs with a few exceptions. Three dogs had mild leukocytosis (15.1 × 10^6^, 15.4 × 10^6^, 16.2 × 10^6^/l; reference interval 4.9–14.7 × 10^6^/l) and one dog had mild erythrocytosis (9.25 × 10^9^/l; reference interval 6.2–8.9 × 10^9^/l). Two dogs had mild hyperglycemia (6.84, 7.2 mmol/l; reference interval 3.7–6.6 mmol/l) and elevated CRP (52, 57 mg/l; reference interval <20 mg/l). One dog had elevations in each of ALP (2.3 µkat/l; reference interval <1.4 µkat/l), creatinine (136 µmol/l; reference interval <135 µmol/l), phosphorus (2.9 mmol/l; reference interval 0.7–1.9 mmol/l), and bile acids (21 µmol/l; reference interval <20 µmol/l).

Plasma cTnI was analyzed in 15 dogs. The median cTnI value at admission was 0.01 ng/ml (IQR 0.01–0.02 ng/ml). All dogs had plasma cTnI concentrations <0.2 ng/ml.

No significant differences in hematologic and serum biochemistry variables were found between dogs with and without pathologic arrhythmias.

## Discussion

The clinically significant arrhythmias detected in this study were of ventricular origin. Pathologic arrhythmias were detected in 47% of included dogs bitten by *V. berus.* Of these eight dogs, four dogs experienced a significant decrease and three a significant increase in arrhythmic episodes in the first 24–32 h following envenomation. All four dogs that were bit on a limb had pathologic arrhythmias. Otherwise, no significant clinical, hematological or biochemical differences between dogs with pathologic arrhythmias and dogs without were found in this study.

In our study, clinically significant arrhythmias were of ventricular origin, whereas other studies of viper envenomation also report of sinus tachycardia, APCs, ST-depression, and sinus arrest [9, 12]. There was a clear distinction between dogs with normal and pathologic 24-AECG recordings, and in no dog, were the findings equivocal. Of the eight dogs with pathologic arrhythmias, arrhythmic events decreased with time in four dogs. This indicates that the venom’s cardiotoxic effects decreased during the 24 h following the snake bite, judged by the waning of ECG abnormalities. On the contrary, in three dogs with pathologic arrhythmias, arrhythmic events significantly increased with time; which implies myocardial damage worsening over time. In one study of *V. berus*-envenomed dogs examined with standard (short-time) ECGs, clinically relevant arrhythmias were detected at admission, between 6–7 h after the snakebite, but not at 12, 24, or 36 h after the bite in one dog [9]. In four of the dogs of this study, arrhythmias were detected either at 12, 24, or 36 h after the snakebite, but not at admission one to 6 h after the bite [9]. In a similar study of 24 dogs, six dogs had arrhythmias at admission and additionally four had arrhythmias 12–24 h post admission, the time lag from envenomation to admission was not reported [10]. Several factors, such as individual susceptibility, site of snake bite, season, and toxicity and volume of injected venom, might influence the presence or absence and timing of arrhythmias. By using a 24-AECG, in the present study, we were able to quantify all arrhythmic variables in the first 24–32 h, and follow possible variations in arrhythmic episodes after the snakebite, which would have been difficult if we had used multiple repeated short-time ECG examinations.

However, 24-AECG might not be an ideal method for use in clinical practice. Interpreting and analyzing the 24-AECG is made after completion of the recording, which would prevent immediate attention to and treatment of arising arrhythmias. Telemetric ECG equipment could be a valuable alternative clinical tool to monitor the individual snake bitten hospitalized dog.

Forty-seven percent of dogs bitten by *V. berus* included in this study experienced pathologic arrhythmias of abnormal ventricular depolarization. Other studies of viper envenomation report lower frequencies of arrhythmic events compared to our study [6, 7, 9, 10, 12, 17]. In publications of viper-bitten dogs where repeated ECG examinations were not performed, arrhythmias were detected by cardiac auscultation in 2–8% of included dogs [6, 7, 17]. Few studies of dogs bitten by vipers have included ECG examinations at predetermined time intervals of 8 or 12 h, to screen for ECG abnormalities, and in these the incidence of arrhythmias was 25, 29, and 42%, respectively [9, 10, 12]. Based on these findings, it appears likely that many arrhythmias remain undetected unless snake bitten dogs undergo repeated ECG examinations or 24-AECG. In fact, arrhythmic events were not detected by auscultation in any of the dogs in the present study. In cases of non-sustained VT, arrhythmias might have been missed if the heart was auscultated in periods of normal sinus rhythm. Furthermore, presence of idioventricular rhythms or VT, with heart rate within normal limits for normal sinus rhythm, might potentially explain the disagreement between auscultatory findings and ECG findings in the present study. Because the 24-AECG recordings were analyzed at the termination of the study, and no arrhythmias were detected by auscultation, none of the studied dogs were treated with any anti-arrhythmic medication.

All four dogs bitten on a limb developed pathologic arrhythmias. However, only four dogs were bitten on a limb, and such a potential association needs to be investigated further by studying a larger number of dogs. The prognosis for included dogs in this study was good regardless of area of bite. However, a study of *V. berus*-envenomated dogs suggested that there might be a correlation between limb envenomation and a poor prognosis [8]. Moreover, a study of *V. palaestinae* envenomation in dogs have found limb envenomation and a body weight less than 15 kg to be significantly associated with increased mortality [1]. This may suggest that the volume of venom injected by vipers in relation to the dog’s size is of importance, and that this volume is inadequate to severely affect large dogs. In addition, dogs younger than 4 years had a lower risk of mortality than older dogs [1]. In the present study, all dogs recovered well from the snakebites. As most dogs were bitten on the nose, and the median age and weight 3 years and 22 and 25 kg respectively, their phenotype might have been favorable in regards to outcome. To further investigate a potential association between bite site and pathologic arrhythmias, studies including a greater number of dogs need to be conducted in the future.

In a study of 24 dogs bitten by *V. berus*, five dogs had elevated serum concentrations of cTnI in the absence of ECG abnormalities [9]. All dogs in the present study had low concentrations of plasma cTnI, analyzed using a high-sensitivity assay, at admission. The blood used for this analysis was sampled only a few hours after envenomation, which might have affected these results.

In humans, a rise in cTnI generally occurs 2–3 h after a myocardial injury [25], and release kinetics of cTnI in dogs appear similar [26]. The median time between snakebites and admission for all the included dogs in the present study was 1.8 h. This short time interval gave limited time for venom absorption from the bite site to the general circulation. As a result, there might have been insufficient time for the circulating venom to cause myocardial injury. This indicates that the peak concentrations of cTnI in our study might not have been detected. However, in a study of dogs envenomated by *V. berus*; differences in cTnI concentrations in dogs, with and without arrhythmias, at admission and 12–24 h after admission could not be detected, and 30% of included dogs had increased cTnI concentrations 5–10 days post admission [10]. One possible explanation to these findings is persistent envenomation caused by continuous release of venom from a depot at the area of the bite. This concept has been demonstrated in a study of humans envenomated by pit vipers that experienced recurrent coagulopathy 2–14 days after envenomation [27].

Because cTnI has a half-life in dogs of 1.85 h [28], these findings suggest ongoing myocardial damage for several days. Also, studies of horses bitten by rattlesnakes measured peak concentrations of cTnI 12 h–1 month after envenomation [15]. In our study, most hematological and biochemical variables were within reference ranges. The reason for this could be that the samples were obtained within few hours after the envenomation episode, and the concentrations of some of these variables may have increased if repeated samples were analyzed. Although this was not investigated in our study, other studies have found that concentrations of ALAT, aspartate amino transferase, and creatine kinase increased from the time of admission to 12–24 h after admission in dogs bitten by *V. palaestinae* and *V. berus* [7, 12].

The mode in which viper toxin causes myocardial damage is not entirely clear. Cardiotoxic components increase cellular membrane permeability to ions; resulting in altered fiber excitability damaging the myocardium and cardiac conduction [2]. This may explain the presence of arrhythmias in the absence of elevated cTnI concentrations seen in other studies [9]. In addition, studies suggest that the systemic inflammation caused by the snake bite, as measured by elevated concentrations of CRP, may injure the myocardium [10]. Also, CRP concentrations in snake bitten dogs have been shown to positively correlate with cTnI concentrations at admission, 12–14 h post-admission, and 5–10 days post-admission [6, 10]. In our study, only two dogs had elevated concentrations of CRP at admission, which made statistical analysis to investigate potential associations with cTnI and the presence of arrhythmias impossible.

There are some study limitations. Although 24-AECG analyses were made by the same person, the included dogs were otherwise examined and treated by different veterinary clinicians (using the same study protocol). This makes comparison of subjective variables between different dogs problematic. Because dogs were examined by 24-AECG in the hospital, they were mostly kept in cages. The results of this study might; therefore, not be directly applicable to dogs that are allowed unrestricted exercise. Another weakness of the study was the small number of included dogs. Included dogs were only examined for 24 h, and ECG findings after the first 24–32 h following a viper bite, could therefore not be evaluated in the present study. Only one blood sample for each dog for hematological and biochemical analyses was obtained at admission, and we were thus unable to investigate variations in concentrations of these variables with time after the snake bites. Moreover, three dogs did not have complete ambulatory ECG recordings of 24 h, and samples for cTnI analysis was lacking for two dogs, which may have affected the final analysis. The samples for cTnI analysis, awaiting transport for storage in −80 °C, were stored in −20 °C. Even though studies of cats have shown that storing samples at −20 °C for a maximum time of three, but not four, months is acceptable, this may have slightly altered the plasma concentrations of cTnI [29].

## Conclusions

In this study of viper-envenomed dogs, the clinically significant arrhythmias were of ventricular origin. Forty-seven percent of included dogs suffered from abnormalities of ventricular depolarization based on findings on the 24-AECG. Dogs experienced a variation of patterns in arrhythmic episodes; some had a decrease and others an increase in abnormal ventricular depolarizations during 24–32 h following the snake bite. Based on findings from the present study, envenomed dogs should be monitored with repeated or continuous ECG-recordings, as differentiating dogs that will develop pathologic arrhythmias from those that will not, using clinical findings and hematologic or biochemical variables obtained at admission appears to be difficult. Dogs that have an increase in pathologic arrhythmic events in the first 24-h following envenomation might benefit from prolonged hospitalization to enable optimal monitoring and treatment of cardiac abnormalities.

## Abbreviations

24-AECG: 24-h ambulatory electrocardiography
ALAT: alanine amino transferase
ALP: alkaline phosphatase
APC: atrial premature complex
AV block: atrioventricular block
BPM: beats per minute
CRP: C-reactive protein
cTnI: cardiac troponin I
ECG: electrocardiography
HR: heart rate
IQR: interquartile range
SVT: supraventricular tachycardia
*V. berus*: *Vipera berus*
*V. palaestinae*: *Vipera palaestinae*
VPC: ventricular premature complex
VT: ventricular tachycardia

## Footnotes

1 Ringer Acetat, Baxter Medical AB, Kista, Sweden.

2 Sysmex-XT2000iV, Sysmex Corporation, Kobe, Japan.

3 Roche Cobas C311, Roches Diagnostics GmBH, D-68298 Mannheim, Germany.

4 Access Systems AccuTnI Assay, Beckman Coulter Inc, Fullerton, CA.

5 GE Vivid E9/Logic E9, GE Medical, Milwaukee WI, USA.

6 Ambu®Blue Sensor M REF: M-00-s/50, Ambu AS, Ballerup, Denmark.

7 Trillium Platinum 5000 Forest Medical, East Syracuse, NY.

8 Trillium Platinum version 4.32 software, Forest Medical, East Syracuse, NY.

9 JMP, version 11.0.0, SAS Institute Inc. Cary, NC.
